# Patterned Metal/Polymer Composite Film with Good Mechanical Stability and Repeatability for Flexible Electronic Devices Using Nanoimprint Technology

**DOI:** 10.3390/mi10100651

**Published:** 2019-09-27

**Authors:** Xu Zheng, Qing Wang, Jinjin Luan, Yao Li, Ning Wang

**Affiliations:** Institue of Nano Engineering, College of Civil Engineering and Architecture, Shandong University of Science and Technology, Qingdao 266590, China

**Keywords:** patterned metal/polymer composite, mechanical properties, interfacial adhesion properties, repeatability, nanoimprint technology

## Abstract

Mechanical stability and repeatability are significant factors for the application of metal film flexible electronic devices. In this work, patterned metal/polymer composite films with good mechanical stability and repeatability were fabricated through nanoimprint technology. The mechanical properties characteristic of metal/polymer composite films were exhibited by resistance change (Δ*R*/*R*_0_) after cyclic tension and bending loading. It was found that the Δ*R*/*R*_0_ and error line of patterned metal/polymer composite film was far lower than the other control groups for repeated experiments, which indicates that patterned metal film has excellent mechanical properties and repeatability. The double cantilever beam method was employed to measure the interfacial adhesion properties of composite films. The average interfacial adhesion of patterned metal/polymer composite films is shown to be over 2.9 and 2.2 times higher than that of metal film deposited on bare polymer and metal nanowire-treated polymer substrates, respectively.

## 1. Introduction

Recently the application of metal/polymer composite films in a flexible strain sensor [1,2], human motion sensor [3,4], microelectronics [5] and film solar cells [6,7] has attracted the attention of researchers. Thus, metal thin films [8,9], metal nanowires [10,11], and graphene [12,13] fabricated on flexible polymers are being generally applied to electronic devices. However, it has been found that the surface of the metal film will trigger irregular cracks under external stresses because of the different elastic modulus in the materials [14,15]. Moreover, delamination and cracks in metal/polymer composite films can easily emerge under cyclic loading because of interfacial bonding failure [16]. This leads to a loss of durability, stability and sensitivity for the device. In addition, the performance of an electronic device obtained by the existing metal film preparation method is unstable under load due to the randomness of crack propagation. Therefore, enhancing the mechanical stability and repeatability of metal/polymer composite films is urgent in order to improve flexible and stretchable electronic devices.

To optimize the problems of interfacial separation and surface cracks, researchers have previously explored various methods. The growth, interfacial adhesion, tensile and flexural properties of composite films are explored in detail through theoretical and experimental methods [17,18,19]. Increasing the roughness of the interface is a method to improve the interfacial adhesion, such as plasma [20,21] and grinding treatment [22] for the polymer substrate. Although this method can increase the interfacial adhesion, it cannot solve the mechanical properties and irregular cracks of metal layer under external load conditions. Moreover, metal nanowires were applied instead of metal films to fabricate flexible devices [23,24,25]. The film formed by metal nanowires will break the circuit and cause the device to be destroyed under cyclic loading conditions. After that, the researchers deposited a metal film on the pre-stretched polymer surface to form the metal/polymer bilayer film with an irregular groove pattern after unloading to improve the mechanical properties [26,27,28]. The surface groove pattern of this method is irregular and cannot be reproduced, and the performance of the device is also unstable. More importantly, because of the instability and randomness of the metal composite materials in the above investigations, no repeated experiments have been conducted, which has made it difficult to apply the devices prepared in the research to industrial production. Nanoimprint lithography (NIL) is a method to prepare regular periodic nano patterns [29,30,31]. Using NIL-fabricated regular periodic grating patterns, repeated experiments can be carried out to study the interfacial adhesion and mechanical properties [32,33,34]. The objectives of this research are to investigate the interfacial adhesion and mechanical properties for metal/polymer composite film, and to fabricate the composite film with stable and repeatable properties through NIL.

In this paper, metal/polymer composite film with regular periodic grating patterns is fabricated to improve the interfacial adhesion and mechanical properties using NIL. The mechanical properties of the composite film were investigated by measuring the surface resistance change (Δ*R*/*R*_0_) for initial state and cyclic loads. A double cantilever beam (DCB) fracture mechanics testing method was used to measure the adhesion energy between Ag thin film and polydimethylsiloxane (PDMS) substrate. The experiments for mechanical properties were explored on multiple repetitive tests to explore the stability and repeatability. The highly increased contact area of patterned metal/polymer composite film enhances mechanical durability and repeatability of the flexible devices from cyclic loadings, which provides a possibility for industrial application.

## 2. Experimental Section

### 2.1. Fabrication of Metal/Polymer Composite Films

**Figure 1:**
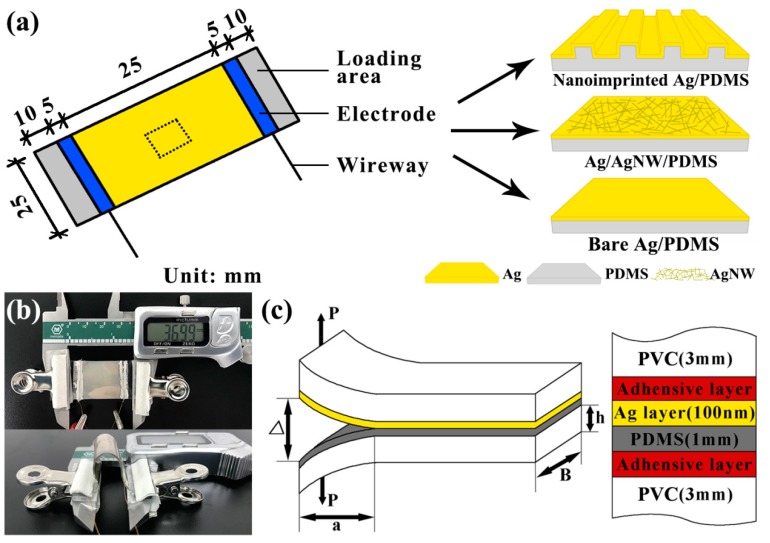
(**a**) Schematic diagram of fabricated samples. (**b**) Photograph of the experimental setup for measuring the Δ*R*/*R*_0_. (**c**) Schematic diagram of double cantilever beam (DCB) test.

The three samples of Ag thin film deposited on bare, Ag nanowires (AgNWs)-treated and nanoimprinted PDMS substrates were used to investigate the performance of composite films. The PDMS (weight ratio of 10:1) was spin-coated on a periodic line array mold (pitch = 200 nm and line width = 100 nm) at a speed of 500 rpm, and reversal imprinted under a pressure of 25 bar at 80 °C for 3 h in vacuum-drying oven through NIL [35,36]. The nanoimprinted PDMS was obtained after demolding from the patterned mold. Similarly, the bare PDMS was fabricated through spin-coating on the bare polycarbonate substrate at a speed of 500 rpm, and cured in a vacuum drying oven. The AgNWs-treated PDMS substrate was prepared by ethanol solution of AgNWs (0.5 mg/mL) coating on bare PDMS through the dip coating method. Then, the Ag thin film (100 nm thickness) was deposited on the patterned, AgNWs-treated and bare PDMS substrates at the same time through vacuum thermal evaporation (Vnano, VZZ-300, Beijing, China). The deposition condition (deposition current = 170 A) and the cooling condition (PDMS substrates were fixed on the cooling plate) were employed for fabricating the samples. The size of fabricated samples is shown in Figure 1a. The right-hand side of Figure 1a shows the enlarged view of the three samples at the dotted line.

### 2.2. Surface Characterization

The surface structures of metal/polymer composite film were obtained by a scanning electron microscope (SEM; Hitachi, S-4800, Tokyo, Japan). Surface resistances were measured by a multimeter. As shown in Figure 1a, copper wires were connected to the Ag film using an electrode (silver paste), and the other end of the copper wires were connected to the multimeter (UNI-T, UT890C+, Dongguan, China). Both ends of the sample were the loading regions. Figure 1b shows the setup used to measure the resistance for the sensor under different strain and bending radius. The sensor was secured to the vernier caliper by two insulating jigs. The appropriate strain and bending radius was achieved by moving the vernier caliper. The resistance was measured by the multimeter, which was connected to the alligator clip and the copper wires.

### 2.3. Adhesion Testing

Interfacial fracture energy is the energy that causes delamination of the layers at the interface. For multilayer materials, delamination will occur when strain energy release rate (*G*) is greater than the interfacial fracture energy (*G_c_*). The DCB fracture mechanics testing method was employed to measure the adhesion energy of metal/polymer composite film. The DCB samples were prepared as a sandwich structure between polyvinyl chloride (PVC) substrates in Figure 1c. Then, the DCB samples were loaded in a mode I tension to measure the adhesion energy and the crack lengths through DCB test equipment (Delaminator Adhesion Test System; DTS Company, Menlo Park, CA, USA). The crack length, *a*, and the applied strain energy release rate, *G*, can be calculated by [37,38]:

(1) $$a={\left(\frac{d\Delta }{dP}\times \frac{BE^{\prime }h^{3}}{8}\right)}^{1/3}-0.64h$$

(2) $$G_{c}=\frac{12P_{c}^{2}a^{2}}{B^{2}E^{\prime }h^{3}}{\left(1+0.64\frac{h}{a}\right)}^{2}$$

where *d*Δ/*dP* is the sample compliance. As shown in Figure 1c, Δ is the total displacement of the beam ends, *P* is the applied load, *B* is the sample width, *E’* is the plane-strain modulus of the beam, *h* is the height of the PVC substrate, *P_c_* is the critical load at which crack growth occurs, and *G_c_* is the interfacial fracture energy. All *G_c_* testing was conducted at ~20 °C and ~35% relative humidity inside the laboratory’s air environment.

## 3. Results and Discussion

### 3.1. Morphology of Ag/Polydimethylsiloxane (PDMS) Composite Films

**Figure 2:**
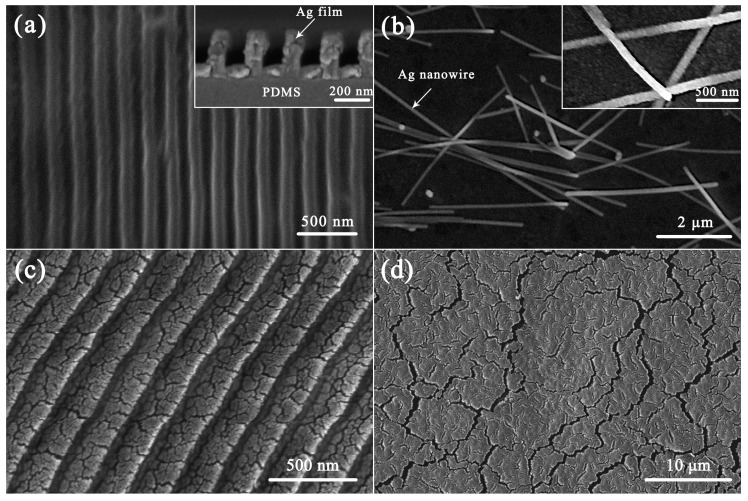
The scanning electron microscope (SEM) images of the fabricated composite films for the initial state: (**a**) Ag/nanoimprinted polydimethylsiloxane (PDMS) and (**b**) Ag/AgNW-treated PDMS. The SEM images of the fabricated composite films after cyclic loading: (**c**) Ag/nanoimprinted PDMS and (**d**) bare Ag/PDMS.

Figure 2 shows the scanning electron microscope (SEM) images of Ag/PDMS composite films under initial state and after cyclic loading. The top-view SEM image of the fabricated Ag/nanoimprinted PDMS in Figure 2a clearly shows the grating structure with a period of 200 nm. The sectional view of Figure 2a can be seen that the mold is completely filled and the Ag film has a thickness of 100 nm. The results show that the mold patterns were perfectly transferred to the film surface through reversal imprint, and the Ag film covers the PDMS surface completely. Figure 2b shows the Ag film deposited on AgNWs-treated PDMS, and the AgNWs were widely distributed on the PDMS substrate. The AgNWs used in this work have a diameter of ≈90 nm and a length of ≈8 μm. In addition, the AgNWs were embedded in the PDMS substrate which enhanced the adhesion strength of the interface.

As shown in Figure 2c, irregular micro-cracks occurred in the horizontal part of the patterned composite film after cyclic loading. Compared with patterned composite film, Figure 2d shows the irregular micro-cracks on the bare composite film. It can be seen that the surface cracks of the bare composite film are much larger than that of patterned composite films. With the patterned composite film stretching, this process can be divided into two stages: film expansion and crack propagation. When the strain increases, the patterned metal film will be spread with the stretched substrate, which is still a continuous metal surface with few cracks. As strain continues to increase, the Ag film will produce small cracks in the weak place. The failure modes of patterned Ag/PDMS composite film can be divided into two modes: the corner mode and the horizontal mode. The corner failure will occur when the thickness of the metal layer at the corner is very weak. The horizontal failure will occur when the thickness of the metal layer at the corner is similar to that at the horizontal part. The reason for this phenomenon is that the corner of the pattern can be expanded with the increase of strain, while the horizontal part of the pattern is more vulnerable to damage under tension.

### 3.2. Tensile Property

**Figure 3:**
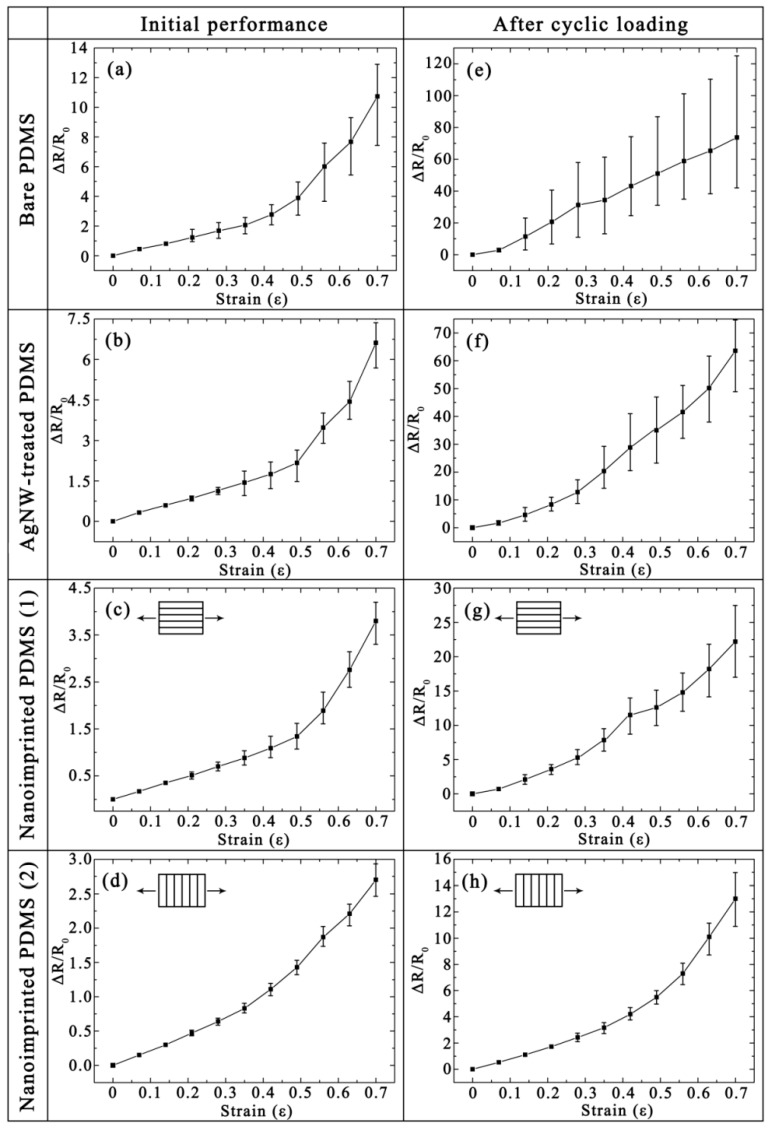
Measured strain-dependent relative Δ*R*/*R*_0_ of Ag thin film on the bare PDMS, Ag nanowire (NW)-treated PDMS and nanoimprinted PDMS substrates under initial state (**a**–**d**) and after cyclic loading (**e**–**h**).

As shown in Figure 3, the tensile properties of Ag thin film on different substrates were evaluated by measuring the Δ*R*/*R*_0_ of metal thin films under initial state and after cyclic loading (1000 cycles with strain of 0.5). To explore the stability and repeatability, the experiments for mechanical properties were explored on six repetitive tests. The values in this figure are the average of six groups of data, and the error lines are the maximum and minimum values of the measured values. The initial sheet resistances (*R*_0_) of Ag films on the bare, AgNWs-treated and nanoimprinted PDMS substrates are 9.6, 6.2, and 14.3 Ω under initial state (Figure 3a–d), respectively. The *R*_0_ of Ag film on AgNWs-treated PDMS is lowest among the other samples because the AgNWs provided as soft conductive bridges improve the stretchability and conductivity of the Ag film. The *R*_0_ of Ag film on nanoimprinted PDMS substrate is highest because the Ag films on the nanoimprinted PDMS surfaces are thinner than other samples due to the vertical deposition of Ag film during the vacuum thermal evaporation process. In addition, the Δ*R*/*R*_0_ of all samples increases with the strain increases. As shown in Figure 3, the Ag film on nanoimprinted PDMS has the smallest Δ*R*/*R*_0_ and error range, and these values of bare PDMS are the largest as the strain increases. This rule is more significant after cyclic loading.

The *R*_0_ of Ag films on the bare, AgNWs-treated, nanoimprinted (parallel direction) and nanoimprinted (vertical direction) PDMS substrates are 19.4, 10.5, 24.6 and 18.7 Ω after cyclic loading (Figure 3e–h), respectively. The *R*_0_ change rate of Ag films on a bare PDMS substrate is the highest (≈102%), which indicates that there are many cracks caused by the serious performance damage in this case. The *R*_0_ of Ag film on the AgNW-treated PDMS substrate is increased from 6.2 to 10.5 Ω, indicating that AgNWs have a tensile resistance as soft conductive bridges during the stretching process. The *R*_0_ change rate of Ag films on a nanoimprinted PDMS substrate (vertical direction) is the smallest, which indicates that patterned metal film has excellent tensile properties. More importantly, as the strain increases, the error line of Δ*R*/*R*_0_ for Ag films on bare, AgNWs-treated, nanoimprinted (parallel direction) PDMS substrates are very large (Figure 3e–g). The results show that the stability of these three samples cannot be guaranteed, and it is difficult to apply them as precision samples to industrial production. However, the error line of Δ*R*/*R*_0_ for Ag films on the nanoimprinted PDMS substrate (vertical direction) is far less than the other three samples, as shown in Figure 3h. This shows that Ag film on the nanoimprinted PDMS substrate (vertical direction) has great stability under cyclic tensile load for six repetitive tests compared with other samples. There are few cracks on the surface of the patterned composite films, so the error lines are much smaller than other samples.

### 3.3. Bending Property

**Figure 4:**
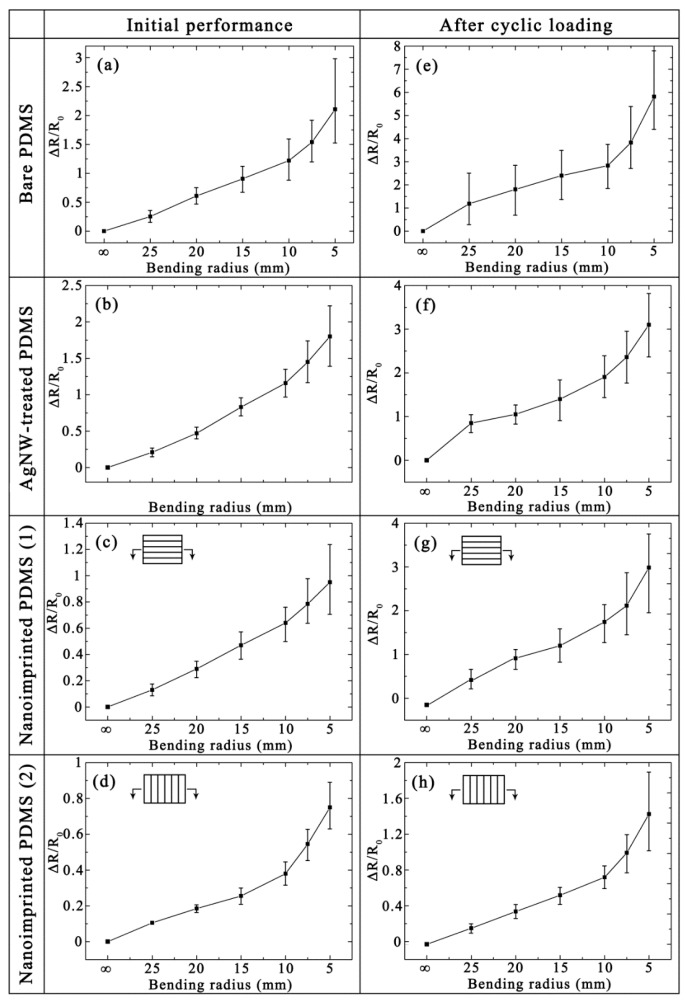
Measured bend-dependent relative Δ*R*/*R*_0_ of Ag thin film on the bare PDMS, AgNW-treated PDMS and nanoimprinted PDMS substrates under initial state (**a**–**d**) and after cyclic loading (**e**–**h**).

Bending resistance is also an important indicator of the mechanical stability for flexible samples. Figure 4 shows the bending properties of Ag thin film on different substrates were evaluated by measuring Δ*R*/*R*_0_ of metal thin films under initial state (Figure 4a–d) and after cyclic loading (Figure 4e–h, 1000 cycles with bending radius of 10 mm). The *R*_0_ of Ag films on different substrates are mentioned above under the initial state. As shown in Figure 4a–d, the error line of Δ*R*/*R*_0_ for Ag films on the bare PDMS is the largest as the bending radius is increasing. Other samples maintain stable performance with a bend radius of less than 10 mm. As shown in Figure 4e–h, the *R*_0_ of Ag films on the bare, AgNWs-treated, nanoimprinted (parallel direction) and nanoimprinted (vertical direction) PDMS substrates are 14.6, 8.9, 19.3 and 17.5 Ω after cyclic loading, respectively. The *R*_0_ increase rate of these four samples are 52.1%, 43.5%, 35.0% and 22.4% compared with the initial state, respectively. The reason for this phenomenon is that the surface cracks of patterned composite films are much smaller than other samples. In this point, the Ag film on nanoimprinted PDMS substrate (vertical direction) has the best bending resistance. It can be seen that the error line of Figure 4f–g gradually increases when the bending radius is less than 20 mm. However, the Ag film on nanoimprinted PDMS substrate (vertical direction) still has excellent stability when the bending radius reaches 10 mm. The excellent bending resistance and stability of the Ag-nanoimprinted PDMS can be mainly attributed to the increase of the surface area with the nanopattering.

### 3.4. Interfacial Adhesion Property

**Figure 5:**
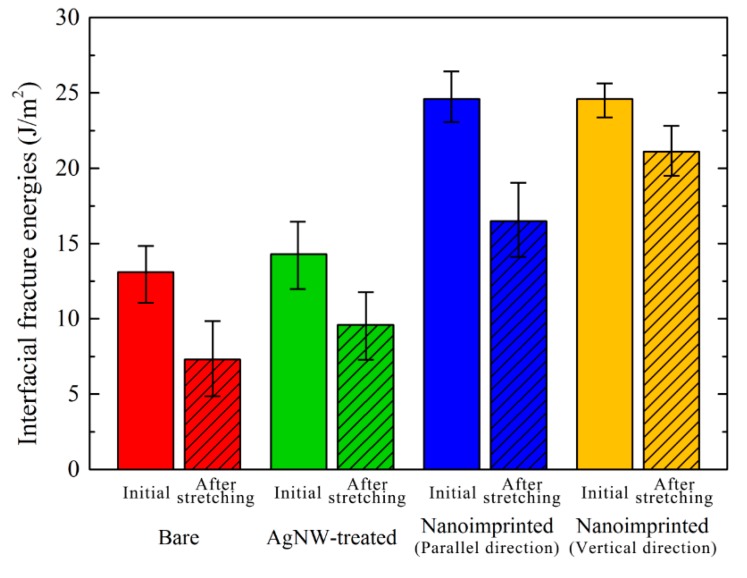
Measured interfacial fracture energies of Ag thin film on the bare PDMS, AgNW-treated PDMS and nanoimprinted PDMS substrates.

As shown in Figure 5, the average values of *G_c_* are 13.1, 14.3 and 24.6 J/m^2^ for the interfaces of Ag/bare PDMS, Ag/AgNWs-treated PDMS and Ag/nanoimprinted PDMS films in the initial stage, respectively. The average values of *G_c_* for Ag/PDMS interfaces are higher than previous research results because of tightly bonded between Ag films and PDMS substrate during the vacuum thermal evaporation process. However, the same evaporation condition would have the same effects on the adhesion properties at the Ag/PDMS interfaces for all samples. The AgNWs-treated PDMS substrate shows slightly higher adhesion energy than that of bare PDMS substrate because of the tensile properties of AgNWs. The interfacial fracture energy of Ag/nanoimprinted PDMS is much higher than that of the other two forms due to the combination of Ag film and nanoimprinted PDMS with the groove pattern increasing the interface contact area. Analysis shows that the process of interface separation is transformed from a mode I fracture to a composite fracture interface of mode I and mode II.

Various *G_c_* for different PDMS substrates show a similar tendency after stretching (strain = 0.5, 1000 cycles) except that their *G_c_* values are lower compared in initial. As shown in Figure 5, their average values of *G_c_* were calculated as 7.3, 9.6, 16.5, and 21.1 J/m^2^ for the interfaces of Ag/bare PDMS, Ag/AgNWs-treated PDMS, Ag/nanoimprinted (parallel direction) PDMS, and Ag/nanoimprinted (vertical direction) PDMS substrates after stretching (strain = 0.5, 1000 cycles), respectively. All the adhesion energies of the Ag/PDMS interfaces are lower after stretching than initially because the stretching load causes cracks on the surface of the Ag film. After cyclic tensile loading, Ag thin films can be easily deboned from PDMS substrates below the *G_c_* due to the increase of cracks on the Ag film. The *G_c_* reduction ratio of AgNW-treated PDMS is significantly smaller than bare PDMS. This phenomenon is due to the irregular cracks on the surface of bare PDMS, and the fact that the AgNWs have the function of resisting stretching. Similarly to the *G_c_* measured in initial, the interfacial adhesion energies of Ag/nanoimprinted PDMS are higher than the values for the other two samples. The measured *G_c_* values for the stretching load parallel to the pattern is slightly lower than the measured values in the vertical direction. When the nanoimprinted PDMS substrates are stretched, the interface of Ag/PDMS is under both shear and tensile stress. When stretched vertical to the pattern, the metal layer can extend with the stretching of the pattern in PDMS. However, in the case of being stretched parallel to the pattern, the metal layer will crack with the stretched PDMS. Therefore, the measured adhesion energy for the load vertical to the line-pattern is much higher than that for the parallel direction.

## 4. Conclusions

The metal thin film deposited on patterned flexible polymer substrate is developed to improve the repeatability and mechanical stability of composite film material through NIL. The nanoimprinted metal/polymer composite film possesses a much smaller resistance change rate and fewer mechanical failures such as crack propagation or delamination after cyclic loading tests as compared to the bare or nanowires-treated polymer substrates. Moreover, the preparation method used in this paper possesses great stability under cyclic tensile or bending loading for repeated experiments. The *R*_0_ change rate of Ag films on the bare, AgNWs-treated and nanoimprinted PDMS substrates are 102%, 69% and 31% after cyclic loading, respectively. From the results of resistance detection and SEM imagines, it can be seen that the surface cracks of patterned composite films were much smaller than other samples. In addition, the interfacial fracture energies of metal/polymer composite films were measured through the DCB fracture mechanics testing method. The average value of *G_c_* (24.6 J/m^2^) for the patterned composite films is about 1.76 times that of other samples, which indicates patterned composite material had the best interface bond strength. The excellent stability of the patterned composite films can be mainly attributed to the increase of the surface area with the nanopatterning. Therefore, this method can be widely used to improve the repeatability and mechanical performance of metal/polymer composite material on flexible electronic devices to provide possibilities for industrial production and prevent potential mechanical failures.
